# A content analysis of online videos containing dietary recommendations for gout and their alignment with evidence-based dietary guidelines

**DOI:** 10.1017/S136898002300160X

**Published:** 2023-10

**Authors:** Kirstie Louise Lamb, Margo E Barker, Anthony Lynn

**Affiliations:** Food and Nutrition Group, Sheffield Hallam University, Sheffield, S1 1WB, UK

**Keywords:** Dietary recommendations, Gout, Evidence-based practice, Patient education material, YouTube

## Abstract

**Objective:**

To assess the alignment of YouTube® videos providing dietary recommendations for gout with evidence-based guidelines targeted at the United Kingdom (UK) population and to establish their quality.

**Design:**

A content analysis of YouTube® videos providing dietary recommendations for gout was undertaken. Videos were categorised by video source. Each video’s dietary recommendations for gout were compared with three evidence-based guidelines for gout, producing a compliance score. Presence of non-guideline advice was assessed. Understandability and actionability were evaluated using the Patient Education Material Assessment Tool for Audio-Visual Materials. Reliability was assessed using an adapted-DISCERN tool and educational quality using the Global Quality Score Five-Point Scale. Differences between video source and continuous variables were assessed using one-way Kruskal–Wallis H tests. For categorical variables, associations were investigated using Fisher–Freeman–Halton tests.

**Setting:**

Online, May–June 2020.

**Participants:**

One-hundred thirty-one videos.

**Results:**

Alignment of videos with evidence-based guidelines was poor (median compliance score 27 % (interquartile range 17–37 %)). Additionally, 57 % of videos contained non-guideline advice. The health professional source group had the fewest videos containing non-guideline advice, but this was only significantly lower than the naturopath group (31 % *v*. 81 %, *P* = 0·009). Almost 70 % of videos were considered poorly actionable and 50 % poorly understandable. Most videos were rated poor for reliability (79 %) and poor to generally poor for educational quality (49 %).

**Conclusions:**

YouTube^®^ videos providing dietary recommendations for gout frequently fail to conform to evidence-based guidelines, and their educational quality, reliability, understandability and actionability are often poor. More high-quality, comprehensive, evidence-based YouTube^®^ videos are required for UK gout patients.

Gout is a painful and debilitating form of inflammatory arthritis with an increasing global prevalence and burden of disease^(1,2)^. In the United Kingdom (UK), approximately 2·5 % of adults were afflicted with gout in 2012, a 64 % increase in prevalence since 1997^(3)^. Gout is associated with numerous co-morbidities, including diabetes, CVD and hypertension, so effective management is crucial^(2,4)^.

Persistently elevated serum urate is a well-recognised risk factor for the development of gout and the reoccurrence of gout flares, because it can contribute to a build-up of monosodium urate crystals within joints^(5)^. Associations have been observed between the consumption of purine- or fructose-rich food and drink, including red meat, beer and sugar-sweetened beverages, and increased uric acid levels and incidence of gout^(6–9)^. In addition to being a risk factor for gout, diet can contribute to its management^(10)^. Consequently, guidelines for the management of gout often include dietary recommendations. These include those targeted at the UK population produced by the British Society for Rheumatology, European League Against Rheumatism and National Institute for Clinical Excellence^(1,11,12)^. Dietary recommendations for gout in these guidelines concur and include restricting most purine- and fructose-rich foods and drinks, limiting alcohol consumption, particularly beer and spirits, remaining hydrated, eating sufficient dairy, and encouraging the consumption of vegetables and fruit^(1,11,12)^.

Despite the availability of evidence-based guidelines, patients may choose alternative sources of information to obtain dietary advice, such as newspapers and online resources^(13–15)^. In the UK, approximately 60 % of the adult population use online sources to access health-related information, including nutrition recommendations^(16)^. Furthermore, an analysis of Internet searches for information on gout reported that the term ‘gout’ is commonly combined with search terms relating to food and diet^(17)^. Online dietary advice may be provided in the form of written, pictorial and/or audio-visual resources. Irrespective of the medium, the information provided by these resources should be easy to understand and consistent with advice from evidence-based sources in order to contribute positively to the self-management of gout^(18–21)^. To our knowledge, no studies have assessed the quality of Internet resources providing dietary recommendations for gout, but analyses of written^(20,22,23)^ and pictorial^(24)^ health advice for the management of gout have frequently reported that resources lack accuracy, provide inadequate information and/or use complicated language which is unsuitable for their intended audience.

Online videos offer an attractive alternative medium through which dietary advice can be obtained. YouTube^®^ (www.youtube.co.uk) is an accessible and popular video-sharing website which may be used for this purpose^(25)^. According to the YouTube^®^ press office, more than 2 billion logged-in users visit YouTube^®^ each month, resulting in an accumulation of over 1 billion hours of watched content every day^(26)^. Despite the popularity of the website, no mandatory editorial or review processes are undertaken during the upload of videos to YouTube^®^, and therefore, the information provided to users may be inaccurate, unreliable, and of poor quality. Indeed, studies of YouTube^®^ videos providing educational information on medical conditions and diseases, including renal disease, kyphosis, rheumatoid arthritis, hypertension, irritable bowel syndrome and severe acute respiratory syndrome (SARS)-CoV-2, have typically reported that a large proportion of videos are inaccurate and/or of poor quality^(27–33)^. A recent study assessing YouTube^®^ videos providing general health information on gout concluded that over 87 % of videos were deemed to be useful and almost 58 % of videos demonstrated high quality^(34)^. However, to our knowledge no studies have reported on the quality and accuracy of YouTube^®^ videos specifically providing dietary recommendations for gout.

The present study therefore aimed to conduct a content analysis to and assess the alignment of videos on YouTube^®^ providing dietary recommendations for gout with evidence-based guidelines. The study also aimed to evaluate the educational quality, reliability, understandability and actionability of these videos and the degree of audience engagement.

## Materials and methods

### Selection of videos

YouTube^®^ (www.youtube.co.uk) was searched in May 2020 for relevant videos using the following search terms: ‘gout diet,’ ‘gout food,’ ‘gout nutrition,’ ‘gout healthy diet’ and ‘gout dietary recommendations.’ Additional search terms were considered, for example ‘foods to eat for gout;’ however, these generated no new videos. Videos were arranged by relevance. As thousands of videos were generated with each search term, it was not possible to analyse every video and so only the first 100 videos for each search term were assessed for eligibility. Videos were excluded if they were not focused on gout (including videos that only referred to hyperuricaemia and/or high uric acid), not focused on humans, did not provide dietary recommendations for gout or had prohibited access. Additionally, videos greater than 20 min in length were excluded, because it has been argued that longer videos may not be viewed in their entirety^(35)^. Videos were not limited to those produced or published by UK sources, but videos were excluded if they were not in English. Where videos were duplicated, the first video was used for analysis and subsequent videos excluded.

### Video characteristics and audience engagement

The source that uploaded each YouTube^®^ video was identified as follows: ‘health professional or organisation,’ ‘naturopath,’ ‘non-medical patient support channel or organisation,’ ‘generic health or diet information channel,’ ‘patient testimonials,’ ‘media’ and ‘other,’ including non-health/diet channels and independent users with no health or medical credentials. The ‘Health professional or organisation’ source group included videos produced by certified dieticians or nutritionists, medical doctors, medical facilities, non-profit healthcare organisations (e.g. the Gout Education Society) and research centres (for example, Arthritis Research Canada). Videos uploaded by individuals who defined themselves as naturopaths or naturopathic practitioners were included in the ‘naturopath’ group. The ‘generic health or diet information channel’ source group included videos uploaded by users with no visible medical credentials on channels that focused on health or diet. ‘Non-medical patient support channel or organisation’ included videos that were uploaded by channels or organisations designed to support patients with gout, but not affiliated with medical professionals or medical organisations. Videos uploaded by patients with gout that explained a personal experience of using diet to manage the condition were included in the ‘patient testimonials’ group. The ‘Media’ source group consisted of videos uploaded by media outlets, such as TV and news channels. The date of upload was noted and used to calculate the number of days since upload. Other descriptive data recorded were the total duration of the video and duration spent discussing dietary recommendations, which were used to calculate the percentage of time spent discussing dietary recommendations. Audience engagement statistics were recorded on 28 May 2020 and included the number of views, ‘likes’ and ‘dislikes’ for a video. The number of views was used alongside the number of days since upload to calculate the view ratio (number of views/number of days since upload). The number of ‘likes’ and ‘dislikes’ were used to calculate the ‘like ratio’ ((number of likes × 100)/(number of likes + number of dislikes)). This ratio was used along with view ratio to calculate the Video Power Index (VPI; (like ratio × view ratio)/100)^(27)^. The number of comments was not recorded, because many comments were found to be unrelated to the corresponding video’s content, for example they contained advertisements for products or websites.

Videos were also classified into one of six topic categories: ‘general diet,’ ‘specific diet,’ ‘specific food or nutrient,’ ‘foods to eat,’ ‘foods to avoid’ and ‘practical guidance.’ ‘General diet’ included videos that covered two or more dietary components. To be included in this category, videos also had to discuss at least one food to avoid *and* at least one food to eat. Videos that discussed only one specific dietary approach, for example ketogenic, Mediterranean, or carnivore diets, were included in the ‘specific diet’ category. ‘Specific food or nutrient’ included videos that only mentioned a single food, food group or nutrient to *either* eat or avoid. ‘Foods to eat’ included videos that had been designed to only discuss foods that were recommended to be eaten. Similarly, ‘foods to avoid’ included videos that had been designed to only discuss foods that were recommended to be avoided. To be included in these two categories, videos had to mention ≥ 2 foods. Videos that had been designed to provide practical recommendations, for example recipes and meal plans, were included in the ‘practical guidance’ category.

### Compliance of videos with guideline recommendations

To evaluate accuracy and comprehensiveness of dietary information for gout, eligible videos were scored against key items of information identified from three evidence-based dietary guidelines for gout to produce a compliance score (online Supplementary Table 1). These guidelines, all targeted at the UK population, were the 2016 updated European League Against Rheumatism Recommendations for the Management of Gout^(11)^, 2017 British Society for Rheumatology Guideline for the Management of Gout^(1)^, and National Institute for Clinical Excellence Gout Diagnosis and Management Guideline^(12)^. Videos addressing multiple dietary components (those with topics identified as ‘general diet’ and ‘specific diet’) were scored against the full thirty items; 1 point was awarded for complete alignment with a guideline item, 0·5 for partial alignment, 0 for not mentioned or discussed and –1 for disagreement with or contradiction to an item. Videos with the topic identified as ‘foods to eat’ were scored against seventeen relevant guideline items only, whilst ‘foods to avoid’ videos were scored against ten relevant guideline items only (online Supplementary Table 1). A method was developed to enable all videos to be analysed together. This consisted of converting compliance scores to a percentage and rating the videos as having low, medium or high compliance with guidelines. Pre-determined arbitrary cut-offs were set to < 33 % for low, 33–66 % for moderate and > 66 % for high compliance. Videos with ‘specific food or nutrient’ and ‘practical guidance’ topics were excluded from this analysis because these videos had been designed to have a narrow focus. If videos contained advice that was not covered by guideline recommendations, this was recorded as ‘non-guideline advice.’

### Quality, reliability, understandability and actionability of videos

The Global Quality Score Five-Point Scale (GQS) was used to rate videos on their overall educational quality and flow of information^(27)^. Videos were rated as ‘poor,’ ‘generally poor,’ ‘moderate,’ ‘good’ or ‘excellent’ quality according to set definitions (online Supplementary Table 2). The reliability of videos was assessed using the adapted-DISCERN tool^(36)^. Videos were scored on their alignment with five reliability criteria, with 1 point allocated for ‘yes’ and 0 points allocated for ‘no’ or ‘unclear’ to produce an overall DISCERN score between 0 and 5 (online Supplementary Table 3). Videos were categorised as having ‘poor’ (score 0–2), ‘fair’ (score 3) or ‘good’ reliability (score 4–5).

The Patient Education Materials Assessment Tool for Audio-Visual Materials (PEMAT-A/V; https://www.ahrq.gov/ncepcr/tools/self-mgmt/pemat.html) was used to assess the understandability and actionability of the dietary information for gout^(37)^. This tool exhibited strong reliability, internal consistency and construct validity during validation^(37)^. Patient education materials are considered to have high understandability when the key points of information can be processed and explained by an audience of diverse health literacy levels and backgrounds. Materials have high actionability when the audience can recognise the steps that they can take based on the information presented. A score of < 70 % is suggested to indicate poor understandability or actionability.

### Statistical analysis

Kolmogorov–Smirnov tests were used to assess the normality of continuous data. Differences between video source and continuous variables were assessed using one-way Kruskal–Wallis H tests. When a statistically significant difference was found, this was followed by a Dunn test with a Bonferroni correction. For categorical variables, associations with video source were investigated using Fisher–Freeman–Halton tests. Statistically significant effects were followed up with post hoc pairwise z-tests with Bonferroni adjustment. Continuous data are reported as medians and interquartile ranges and categorical data as percentages or proportions. The degree of agreement between two authors who assessed the compliance of videos with guideline recommendations was determined using Cohen’s kappa (inter-rater agreement) coefficient of agreement. All analyses were conducted using IBM SPSS Statistics, version 24 (SPSS Inc., Chicago). The critical value for statistical significance was set at *P* ≤ 0·05.

## Results

### Video identification

**Fig. 1:**
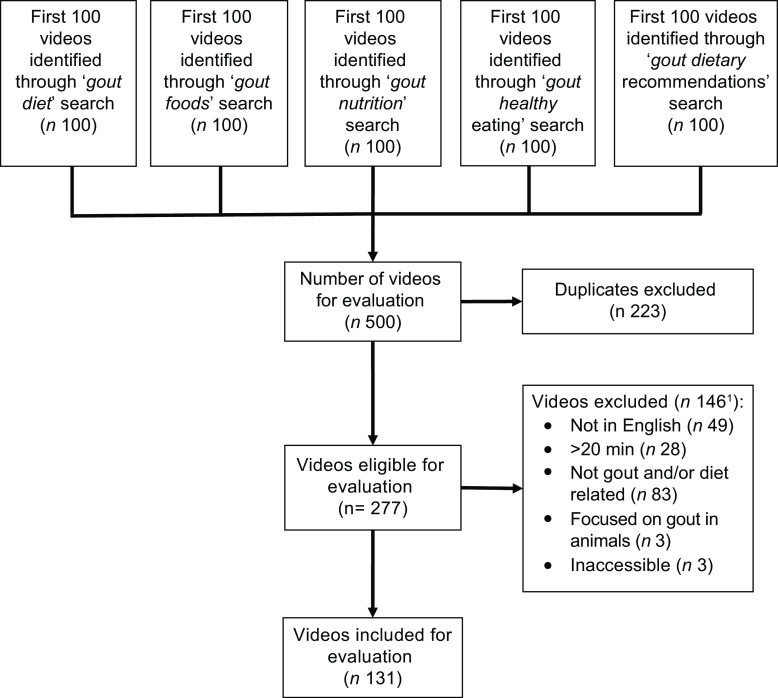
Flowchart of video selection process, including reasons for exclusion of videos. ^1^ Nb. The sum of the video meeting each exclusion criteria is not equal to the total number of videos excluded, because some videos met more than one exclusion criteria

In total, 131 eligible videos were identified from a total pool of 500 videos (first 100 videos for each of the search terms used). The process of video identification is displayed in Fig. 1.

### Video characteristics and audience engagement

The characteristics and audience engagement metrics of videos are displayed in Table 1. The main source of videos was ‘health professional’ (*n* 42/131), followed closely by ‘generic diet and health information channels’ (*n* 36/131). The median number of days since upload was 947, and median duration of videos was 3 min 34 s (*n* 131). Videos had a median of nine views per day since upload, sixty likes and four dislikes. On average, dietary recommendations were discussed for 70 % of the total video time.

**Table 1:** Characteristics and audience engagement metrics of 131 YouTube® videos providing dietary recommendations for gout after grouping by video upload source

		Overall (*n* 131)	Health professional (*n* 42)	Naturopath (*n* 16)	Patient support (*n* 16)	Generic diet/health information channel (*n* 36)	Media (*n* 4)	Patient testimony (*n* 2)	Other (*n* 15)
Time since upload*	Days	947	1287	787	388	930	1098	1176	947
	IQR†	470–1834	598–2560^1^	377–1800^1 2^	229–527^2^	511–1230^1 2^	888–1389^1 2^	1160–1191^1 2^	821–2887^1 2^
Duration* ^,‡^	s	214	194	273	189	261	110	232	219
	IQR	134–345	112–373	170–554	125–204	162–363	67–275	185–277	187–335
Video time specifically discussing diet recommendations*	%	70	54	67	97	76	75	81	63
	IQR	52–86	24–71^1^	60–77^1^	94–97^2^	59–86^1^	61–80^1 2^	74–87^1 2^	48–79^1^
View ratio*	views/day	9·1	16·3	18·8	2·1	17·0	0·5	66·8	14·8
	IQR	1·3–64·8	1·5–60·5^1 2^	3·0–112·4^1 2^	0·7–3·3^1^	3·9–126·6^2^	0·4–0·6^1 2^	35·8–97·7^1 2^	3·8–546·9^2^
Like ratio	%	93	93	95	96	92	95	94	93
	IQR	88–97	87–98	92–97	88–100	86–96	85–100	92–97	84–95
VPI§,*		7·1	10·2	14·4	1·6	15·	0·4	59·9	13·7
	IQR	1·1–56·4	1·3–50·8^1 2 3^	2·3–65·0^1 2 3^	0·5–2·8^1^	0 3·5–118·8^2^	0·3–0·6^1 3^	32·4–87·5^1 2 3^	2·8–514·0^2 3^

Values are displayed as displayed as median (IQR) values.

* Significantly different (*P* < 0·05) across video sources according to one-way Kruskal-Wallis test. Values with different superscript numbers are significantly different from each other at *P* < 0·05 following post hoc Dunn test with Bonferroni correction.

† IQR, interquartile range.

‡ Post hoc tests revealed no differences between video source categories.

§ VPI, Video Power Index.

There were statistically significant differences in days since upload (*P* = 0·022) between video source categories. ‘Patient support’ videos had the lowest number of days since upload at 388 d (229–527 d), and this was significantly lower than the ‘health professional’ videos (*P* = 0·012). Duration was also found to be significantly different across source categories according to the one-way Kruskal–Wallis test (*P* = 0·021), but post hoc tests revealed no differences between categories. All video source categories had like ratios greater than 90 % and there were no statistically significant differences across categories.

The amount of time spent discussing dietary recommendations differed by video source (*P* < 0·001). At 97 % (94–97 %), non-medical patient support channel or organisation (‘patient support’) videos spent the greatest percentage of time discussing dietary recommendations, and this was significantly greater than ‘health professional’ (*P* < 0·001), ‘naturopath’ (*P* = 0·007), ‘generic diet and health information channels’ (*P* = 0·006) and ‘other’ (*P* = 0·001) videos.

There were statistically significant differences in view ratio according to video source category (*P* = 0·001). View ratio was highest in the ‘patient testimony’ category, but there were only two videos in this group. ‘Generic diet and health information channel’ and ‘other’ categories were shown to have significantly higher view ratios than the ‘patient support’ category (*P* = 0·008 and *P* = 0·027, respectively).

VPI differed significantly by video source (*P* < 0·001) and was also highest for the ‘patient testimony’ category. The ‘generic diet and health information channel’ category, which had the second highest VPI at 15·0 (3·5–118·8), was significantly higher than the ‘media’ (*P* = 0·017) and ‘patient support’ categories (*P* = 0·001). The ‘other’ video source category also had a significantly higher VPI score than the ‘patient support’ category (*P* = 0·018).

Overall, approximately 65 % (*n* 82) of the videos discussed multiple dietary components. Specific diets or dietary approaches were the focus of 12 % (*n* 10) of these, equivalent to 8 % of all videos analysed, and included low carbohydrate, ketogenic, Mediterranean, vegetarian and carnivore diets. Meanwhile, 19 % of videos (*n* 25) concentrated on a specific food, food group or nutrient, whilst 16 % (*n* 21) focused on either food to avoid or food to eat. Only 2 % of videos (*n* 3) were designed to solely provide practical guidance or advice such as meal plans and recipes.

### Compliance of videos with guideline recommendations

The distribution of guideline items discussed in the 131 videos is displayed in Table 2. The three most popular items covered in the YouTube^®^ videos were ‘avoid excessive meat intake’ (70 %, 92/131), ‘avoid excessive alcohol intake’ (57 %, 75/131) and ‘avoid excessive seafood intake’ (55 %, 72/131). The three least common items discussed were ‘avoid crash dieting/weight loss should be gradual’ (3 %, 4/131), ‘encourage folate intake’ (2 %, 3/131) and ‘fluid/water intake is especially important for those with kidney stones’ (2 %, 3/131).

**Table 2:** Guideline items covered across 131 YouTube^®^ videos providing dietary recommendations for gout. Values displayed as the total number and percentage of sample that covered each item

Guideline item	*n* videos covering item (% total sample)	%
Avoid excessive meat intake	92	70
Avoid excessive alcohol intake/drink alcohol sensibly	75	57
Avoid excessive seafood intake	72	55
Encourage a diet high in vegetables	69	53
Encourage fruit consumption	61	47
Avoid excessive purine intake	59	45
Avoid sugar-sweetened drinks	46	35
Encourage fluid/water intake to prevent dehydration (> 2 l)	45	34
Encourage (low-fat) dairy consumption	44	34
Encourage a diet high in fibre	41	31
Encourage a diet low in added sugar/avoid excessive sugar consumption	39	30
Avoid excessive consumption of beer	39	30
Encourages consumption of cherries	38	29
Consumption of vitamin C may be beneficial	35	27
Taking prescribed gout medication is still important	28	21
Weight loss should be encouraged if appropriate	26	20
Avoid fructose-rich foods	25	19
Coffee consumption may reduce recurrent gout flares	23	18
Encourage diet low in fat	22	17
Encourage skimmed milk consumption	21	16
Encourage regular exercise	17	13
Encourage low-calorie/low-fat yoghurt consumption	16	12
Moderate intake of purine-rich vegetables okay/does not increase risk	16	12
Encourage consumption of soybeans and other vegetable protein sources	15	12
Moderate wine intake (2 glasses/d) acceptable/does not increase risk	10	8
Avoid excessive consumption of spirits	9	7
Reduce orange and apple juice consumption	6	5
Weight loss should be gradual/avoid crash dieting	4	3
Encourage folate intake	3	2
Fluid/water intake is especially important for those with kidney stones	3	2

The dietary advice contained in YouTube^®^ videos typically failed to align with guideline recommendations resulting in a median compliance score of 27 % (interquartile range 17–37 %). Almost three-quarters of videos were rated low for their compliance and only 5 % of videos were rated high (Table 3). There were no significant differences in the accuracy and comprehensiveness of recommendations across video source categories, as assessed by compliance scores (H (5) = 10·07, *P* = 0·073) or the distribution of compliance ratings (Fishers = 13·21, *P* = 0·128). However, there was a significant difference in the number of videos containing one or more pieces of non-guideline advice across the video source categories (Fishers = 20·44, *P* = 0·001), examples of which included the consumption of apple cider vinegar, dandelions and tree leaves. The ‘health professional’ category had the lowest percentage of videos containing non-guideline advice at 31 %, and this was significantly lower than the ‘naturopath’ video source category at 81 % (*P* = 0·009). The inter-rater agreement between two reviewers who assessed the compliance of videos with guideline recommendations was 93 % with a kappa coefficient of 0·789 (*P* < 0·001).

**Table 3:** Alignment of dietary information for gout provided by 131 YouTube® videos, grouped by video upload source, with key items from dietary guidelines for gout

		Overall (*n* 131)		Health professional (*n* 42)		Naturopath (*n* 16)		Patient support (*n* 16)		Generic diet/health information channel (*n* 36)		Media (*n* 4)		Patient testimony (*n* 2)†		Other (*n* 15)	
		%	*n*	%	*n*	%	*n*	%	*n*	%	*n*	%	*n*	%	*n*	%	*n*
Presence of non-guideline advice, % category (*n* videos)*		57	75	31	13^1^	81	13^2^	75	12^1 2^	61	22^1 2^	50	2^1 2^	100	2	73	11^1 2^
Number of videos analysed for compliance score, *n*‡		103		37		13		9		29		3		0		12	
Compliance score‡																	
%		27		25		23		18		30		18		n/a		32	
IQR§		17–37		17–33		18–30		15–28		21–60		16–18				16–48	
Compliance rating, % category (*n* videos)‡	Low	71	73	76	28	92	12	89	8	55	16	100	3	n/a		50	6
	Mod.||	24	25	22	8	8	1	11	1	31	9	0	0	n/a		50	6
	High	5	5	2	1	0	0	0	0	14	4	0	0	n/a		0	0

Values displayed as number and percentage of videos containing non-guideline advice, median compliance score (%) and percentage and number of videos in each compliance rating category.

* Significant difference between number of videos with non-guidance advice and video source category (*P* < 0·05). Values with differing superscript numbers are significantly different from each other at *P* < 0·05 following post hoc comparison.

† This category was not used in post hoc comparisons because its column proportions were equal to zero or one.

‡ Compliance score and rating (*n* 103): ‘specific food/nutrient’ and ‘practical guidance’ video scores were excluded. Patient testimony videos did not receive a compliance score or rating, because both videos (*n* 2) were within the excluded ‘specific food/nutrient’ topic group.

§ IQR, interquartile range.

|| mod., moderate.

### Reliability, quality, understandability and actionability of videos

Almost 80 % of videos were considered poor in terms of their reliability (adapted-DISCERN). Although the ‘health professional’ source group had the highest percentage of videos rated ‘fair’ or ‘good’ for reliability, there were no significant differences between reliability rating categories (Fishers = 12·43, *P* = 0·320) or in total reliability scores (H (6) = 6·86, *P* = 0·334) between video source categories (Table 4). Almost half of the videos were rated poor to generally poor for educational quality (GQS). Only the ‘health professional’ source group contained a video rated ‘excellent’ for quality. However, the number of videos in each educational quality rating category (Fishers = 25·85, *P* = 0·361) and total educational scores (H (6) = 6·36, *P* = 0·384) did not differ significantly between video source categories. Only 50 % (*n* 66) and 22 % (*n* 29) of the videos had ratings at 70 % or above for understandability and actionability (PEMAT), respectively. Whilst understandability did not vary significantly between video source categories, actionability was significantly higher for videos in the ‘patient support’ category than in the ‘health professional’ category (*P* = 0·016). Within the ‘health professional’ category, five videos were uploaded by dieticians. An exploratory analysis revealed that these five videos had a higher median actionability score when separated from the other 37 ‘health professional’ videos (67 % and 33 %, respectively). However, this difference was not statistically significant (*P* = 0·375).

**Table 4:** Analysis of the reliability, educational quality, understandability and actionability of 131 YouTube® videos providing dietary recommendations for gout after grouping by video upload source

	Overall (*n* 131)		Health professional (*n* 42)		Naturopath (*n* 16)		Patient support (*n* 16)		Generic diet/health information channel (*n* 36)		Median (*n* 4)		Patient testimony (*n* 2)		Other (*n* 15)	
	%	*n*	%	*n*	%	*n*	%	*n*	%	*n*	%	*n*	%	*n*	%	*n*
Reliability of information, assessed using the adapted-DISCERN tool																
Poor	79	104	62	26	88	14	94	15	86	31	75	3	100	2	87	13
Fair	15	19	26	11	6	1	6	1	8	3	25	1	0	0	13	2
Good	6	8	12	5	6	1	0	0	6	2	0	0	0	0	0	0
Educational quality of videos, assessed using the GQS† tool																
Poor	12	16	5	2	19	3	0	0	22	8	0	0	50	1	13	2
Generally poor	37	49	38	16	31	5	56	9	25	9	75	3	50	1	40	6
Moderate	37	49	36	15	50	8	44	7	36	13	25	1	0	0	33	5
Good	12	16	19	8	0	0	0	0	17	6	0	0	0	0	13	2
Excellent	1	1	2	1	0	0	0	0	0	0	0	0	0	0	0	0
Understandability and actionability, assessed using the PEMAT‡ tool, % score (IQR§)																
	% score	IQR	% score	IQR	% score	IQR	% score	IQR	% score	IQR	% score	IQR	% score	IQR	% score	IQR
Understandability	70	60–78	70	60–80	67	58–70	70	60–80	65	60–72	50	49–58	63	62–65	70	67–78
Actionability *	67	33–67	50	33–67^1^	67	58–100^1 2^	67	67–100^2^	67	33–67^1 2^	67	50–69^1 2^	83 ^2^	75–92^1^	67	67–67^1 2^

GQS and adapted-DISCERN scores displayed as percentage and number of videos within each score category. PEMAT scores are displayed as median (IQR).

* Significant difference between actionability score and video source category (*P* < 0·05). Values with different superscript numbers are significantly different from each other at *P* < 0·05 following post hoc comparison.

† GQS, Global Quality Score Five-Point Scale.

‡ PEMAT, Patient Education Materials Assessment Tool for Audio-Visual Materials.

§ IQR, interquartile range.

## Discussion

This study found that dietary information provided by the majority of YouTube^®^ videos analysed complied poorly with evidence-based dietary guidelines for gout. A high proportion of videos were also rated poor in terms of their reliability, educational quality, understandability and actionability and thus may be deemed unsuitable for UK gout patients to use for nutritional advice.

Almost 60 % of the videos analysed provided at least one dietary recommendation that did not align with the three evidence-based guidelines. These aberrant recommendations often focused on the consumption of traditional remedies that have yet to be researched such as apple cider vinegar or others for which only very limited data of efficacy exist, such as raw garlic and celery extracts powder^(38,39)^. Some videos also recommended the avoidance of carbohydrates, yet to our knowledge no studies have reported on the effect of low carbohydrate diets on the management of gout patients. Relatively few studies have focused solely on the accuracy of dietary information provided in YouTube^®^ videos. However, in a content analysis of YouTube^®^ videos on diet and renal disease, 82 % of videos contained misleading information, compared with our finding of 57 %^(28)^. In contrast, studies looking at general health-related information on medical conditions, including rheumatoid arthritis, hypertension, kidney stone disease and more recently gout, have reported much lower percentages (12 – 33 %) of videos containing misleading or inaccurate information^(29,31,32,34)^. The high prevalence of misleading information in videos providing dietary advice may reflect the fact that diet is a controversial area of health management, susceptible to many differing opinions, myths and misconceptions^(40,41)^.

In addition to providing dietary recommendations that did not align with guidelines, many videos only discussed a few dietary components. These factors contributed to the low overall compliance score of 27 % across all video source categories. The most frequently discussed diet topics were reducing or restricting meat, alcohol and/or seafood intake. These dietary components have been consistently associated with increased uric acid levels and risk of gout, so it is encouraging that this advice was provided in more than 50 % of videos^(6,7,11)^. Nevertheless, several other important dietary factors were rarely addressed. For example, less than 1/3 of videos specifically discouraged excessive beer consumption, yet associations between beer consumption increased uric acid levels and risk of gout are convincing^(42,43)^. Shulten *et al.* (2009) observed that the beer-drinking behaviour of gout patients was frequently inconsistent with evidence-based guidelines, possibly caused by a lack of patient awareness^(10)^. Videos should therefore discuss as many evidence-based dietary factors as possible to equip patients with the knowledge needed to optimise the non-pharmaceutical management of their condition.

Although there were no statistically significant differences in overall compliance scores between video source groups, videos uploaded by naturopaths were found to be the main contributors of ‘misleading’ videos, with over 80 % of these videos containing at least one piece of non-guideline advice. This is a common finding; misleading and/or erroneous information was identified in 78 % of Internet resources produced by naturopaths on renal diets^(28)^. Furthermore, 92 % of videos providing information on rheumatoid arthritis have previously been found to promote unscientific therapies, which included naturopathic therapies^(29)^. Naturopathic practitioners favour the use of complementary and alternative medical therapies such as herbs in their practice, but these often have insufficient scientific evidence to support their inclusion in evidence-based guidelines^(44)^. In contrast, less than one-third of videos within the ‘health professional’ category provided advice outside of the guidelines. It is encouraging that almost a third of videos were uploaded by health professionals, because this increases the likelihood of patients encountering information that is consistent with guidance provided by their healthcare team.

Video Power Index scores indicated that videos from the ‘health professional’ category were not favoured over videos from other sources, suggesting that individuals do not actively seek out videos from reliable sources. This is consistent with a content analysis of YouTube^®^ videos providing information on dialysis; engagement was found to be lowest with videos that were identified by two physicians as useful^(45)^. Whilst some viewers may be unaware of the inaccuracies and inconsistencies of information provided in YouTube^®^ videos, or the credibility of the source of this information, others may choose to use complementary and alternative treatments for their condition and actively seek videos containing related information. Indeed, Chan *et al.* (2014) reported that 23·9 % of patients with gout used complementary and alternative medicine for their condition^(46)^. However, where information obtained by patients conflicts with that provided by their healthcare team, this can increase the risk of inadequate self-management of chronic medical conditions^(21,47)^. To reduce this risk for individuals with gout, it is vital that patients are guided by their healthcare team towards videos that are consistent with evidence-based guidelines and are educated on the sources of information to avoid.

Irrespective of source, most YouTube^®^ videos analysed displayed poor educational quality and reliability. This finding is consistent with other studies assessing the quality and/or reliability of YouTube^®^ videos providing health information^(27,28,30)^. Median understandability was 70 %; this is the lowest possible threshold for being classed as understandable, as defined by Shoemaker *et al.* (2014)^(37)^ and suggests that the structure and language of many videos could be improved to make them more appropriate for their target audience Most videos were poorly actionable, further reinforcing the unsuitability of these videos. Actionability was found to be significantly higher in ‘patient support’ videos than ‘health professional’ videos. Additionally, ‘health professional’ videos spent a significantly lower proportion of time discussing dietary recommendations than ‘patient support’ videos. These findings could be explained by differing target audiences and overall purpose of videos; by definition, ‘patient support’ videos are more likely to be targeted explicitly at patients with gout and designed to help them manage their condition, whilst ‘health professional’ videos may be aimed at educating a wider target audience, including other healthcare professionals and those with no prior knowledge of gout. Lambert *et al.* (2017) also observed that videos uploaded by medical doctors scored poorly for actionability; however, those uploaded by dietitians scored higher^(28)^. In the present study, only five of the forty-two health professional videos were uploaded by dieticians and so these were initially combined in the ‘health professional’ category. When separated from the other thirty-seven ‘health professional’ videos, the five videos uploaded by dieticians had a higher median actionability score. However, the difference was not statistically significant and both scores remained below the actionability threshold of 70 %.

This study has added to a growing body of literature suggesting that health information from videos on YouTube^®^ is often of poor quality and reliability and inconsistent with national and/or international guidelines^(27–31)^. The UK public need to take great care when selecting YouTube^®^ videos containing dietary recommendations for gout, because a large proportion of available information is inconsistent with the evidence-based advice provided by healthcare teams. A screening process to prevent inaccurate or unreliable information from being uploaded on YouTube^®^ should be considered in the future. Regulation of online health information could also be considered, although the difficulties and limitations of this have previously been recognised^(48)^. For now, however, where YouTube^®^ is a preferred source of information for patients, it would be beneficial for healthcare teams to assist in identifying appropriate videos for their patients to watch. There is also a need for health professionals to follow evidence-based guidelines and to consider actionability and educational quality when producing health information videos for upload onto YouTube^®^. Additionally, a peer-review process could further improve the accuracy of such videos.

The present study has several limitations, including its cross-sectional design. This study was undertaken at the beginning of the COVID-19 pandemic in the UK. As a result of reduced face-to-face contact with health professionals, an increased number of individuals may have accessed online resources, such as YouTube^®^, for health information during this time^(49)^. As YouTube^®^ video engagement metrics are constantly changing, future research could look at measuring the change in engagement metrics for videos over time. It is also acknowledged that search results may vary between YouTube^®^ users, as YouTube^®^ can consider search and watch history when organising search results by relevance. In the current study, a YouTube^®^ account with no previous search history was utilised to eliminate this influence, but this may not be the case for many other users. Furthermore, YouTube^®^ also considers aggregate user engagement signals when determining video relevance and so it is possible that some relevant videos may not have been included in the first 100 videos generated for each search term.

Videos were also predominantly uploaded by non-UK sources. Thus, content scores for these videos may have differed slightly had they been scored against dietary guidelines for gout from their respective countries. However, whilst there may be small differences in guideline recommendations, the key components of the guidelines used in the present study, such as limiting consumption of high-purine meat, seafood, and alcohol, do not appear to differ substantially across countries^(50)^. Meanwhile, most of the dietary components classified as ‘non-guideline advice’ in this study, such as consuming apple cider vinegar, also do not appear in other countries’ evidence-based guidelines.

Videos greater than 20 min in length were excluded from the analysis, in line with a previous study of health information videos^(33)^. It is possible that videos exceeding 20 min duration could have time to cover a greater number of dietary recommendations and thus may have produced higher compliance scores than those under 20 min. However, Robitza et al (2020) have shown that longer videos tend not to be watched in their entirety, which could limit their usefulness^(35)^.

Finally, the present study was not designed to measure the impact of using YouTube^®^ videos from different sources on the self-management of gout. It could be informative to address this in future research.

A strength of this study is the number of search terms utilised, as many previous YouTube^®^ video content analysis studies have limited searches to 2–3 terms^(28,29,33)^. In the present study, additional videos were identified as search terms increased to 5, but then additional search terms did not generate any further videos.

### Conclusions

In conclusion, it is evident that YouTube^®^ videos on diet and gout often fail to provide important information contained in evidence-based dietary guidelines targeted at the UK population. Instead, videos frequently include non-evidence-based information. Dietary information provided by these videos is also commonly poor in terms of educational quality, reliability, understandability and actionability. These factors may result in poor self-management of gout by patients who use YouTube^®^ videos as a source of information. Healthcare teams in the UK should signpost appropriate and evidence-based sources of information to their patients. Furthermore, health professionals should ensure that the videos they produce are evidence-based and high in educational quality and actionability. Future research should aim to measure the impact of YouTube^®^ video usage from different upload sources on the self-management of gout.

## Supplementary material

For supplementary material accompanying this paper visit https://doi.org/10.1017/S136898002300160X. click here to view supplementary material
