# Supplementary material for: A content analysis of online videos containing dietary recommendations for gout and their alignment with evidence-based dietary guidelines
Source: Public Health Nutr. 2023 Aug 14;26(10):2014–25. doi: 10.1017/S136898002300160X (PMC10564598; doi:10.1017/S136898002300160X)
Supplement: Supplementary file 1 [file S136898002300160Xsup001.docx]

**Supplementary table 1. Dietary recommendation items from British Society for Rheumatology (BSR), European League against Rheumatism (EULAR) and National Institute for Clinical Excellence (NICE) guidelines for the management of gout that YouTube**® **videos were scored against to produce compliance scores**

| Full 30 items from guidelines | 17 items that ‘Foods to eat’ videos were scored against | 10 items that ‘Foods to avoid’ videos were scored against |
| --- | --- | --- |
| Avoid excessive meat intake |  | **X** |
| Avoid excessive alcohol intake/drink alcohol sensibly |  | **X** |
| Avoid excessive consumption of beer |  | **X** |
| Avoid excessive consumption of spirits |  | **X** |
| Avoid excessive seafood intake |  | **X** |
| Avoid excessive purine intake |  | **X** |
| Avoid fructose-rich foods |  | **X** |
| Avoid sugar-sweetened drinks |  | **X** |
| Reduce orange and apple juice consumption |  | **X** |
| Encourages diet low in sugar/Avoid excessive sugar consumption | **X** | **X** |
| Encourage a diet high in vegetables | **X** |  |
| Encourage fruit consumption | **X** |  |
| Encourage fluid/water intake to prevent dehydration (>2 litres) | **X** |  |
| Encourage (low-fat) dairy consumption | **X** |  |
| Encourage a diet high in fibre | **X** |  |
| Encourages consumption of cherries | **X** |  |
| Consumption of vitamin C may be beneficial | **X** |  |
| Coffee consumption may reduce recurrent gout flares | **X** |  |
| Encourage diet low in fat | **X** |  |
| Encourage skimmed milk consumption | **X** |  |
| Encourage regular exercise |  |  |
| Encourage low-calorie/low-fat yoghurt consumption | **X** |  |
| Moderate intake of purine-rich vegetables okay/does not increase risk | **X** |  |
| Encourage consumption of soybeans and other vegetable protein sources | **X** |  |
| Moderate wine intake (2 glasses/day) acceptable/does not increase risk | **X** |  |
| Encourage folate intake | **X** |  |
| Fluid/water intake is especially important for those with kidney stones | **X** |  |
| Taking prescribed gout medication is still important |  |  |
| Weight loss should be encouraged if appropriate |  |  |
| Weight loss should be gradual/avoid crash dieting |  |  |
